# First molecularly confirmed case of canine thelaziosis due to *Thelazia callipaeda* infection in Estonia

**DOI:** 10.1186/s13028-026-00864-7

**Published:** 2026-04-04

**Authors:** Kristi Sisask, Maare Mõtsküla, Urmas Saarma

**Affiliations:** 1 Small Animal Clinic, The Institute of Veterinary Medicine and Animal Sciences, Estonian University of Life Sciences, Kreutzwaldi 62, Tartu, 51014 Estonia; 2 Department of Zoology, Institute of Ecology and Earth Sciences, University of Tartu, J. Liivi 2, Tartu, 50409 Estonia

**Keywords:** Canine, Eye worm, Milbemycin oxime, Ocular thelaziosis, Vector-borne zoonosis

## Abstract

**Background:**

*Thelazia callipaeda* is a zoonotic vector-borne nematode primarily affecting carnivores, lagomorphs, and humans. Over the past decades, the parasite has expanded across Europe. This report presents the first molecularly confirmed case of canine thelaziosis in Estonia. The findings highlight the urgent need for increased awareness of *T. callipaeda* among veterinarians, human medical professionals, and pet owners, as this parasite poses an important public health concern.

**Case presentation:**

A 6-year-old spayed female Swiss White Shepherd, with a recent travel history to several European countries, including the Baltic countries, Poland, the Czech Republic, Austria, Italy, Switzerland, Liechtenstein, Germany, and Sweden, was presented to the Small Animal Clinic of Estonian University of Life Sciences with unilateral ocular discharge and hyperaemia. Ophthalmic examination revealed the presence of motile, threadlike nematodes in the left eye. The treatment involved manual removal of nematodes and oral administration of milbemycin oxime/praziquantel twice with 1 week interval in combination with topical lubrication. Follow-up examinations revealed significant improvement in ocular health, with no further signs of infection. The nematodes were identified as *T. callipaeda* based on molecular analysis.

**Conclusions:**

This case highlights the importance of obtaining a comprehensive travel history in veterinary ophthalmology and emphasizes the emerging risk of *T. callipaeda* in non-endemic regions, especially with increased pet travel. Early diagnosis and appropriate treatment are crucial for successful management. Preventive measures, such as antiparasitic treatment before and during travel, are essential to reduce the risk of transmission. The growing concern of *T. callipaeda* spread is further supported by previous ecological niche model predictions, suggesting the potential for the vector’s geographic expansion across Europe.

## Background

*Thelazia callipaeda*, commonly known as the “oriental eyeworm”, is a zoonotic vector-borne nematode first described in Asia in the early 20th century. In recent decades, *T. callipaeda* has been increasingly reported across Europe, including in Turkey, Romania, Bulgaria, Greece, Serbia, Hungary, Slovakia, Poland, the Czech Republic, Austria, Croatia, Bosnia and Herzegovina, Germany, Switzerland, Italy, France, Belgium, Spain, Portugal, and the United Kingdom [1–7]. The parasite infects a range of domestic and wild carnivores (e.g., dogs, cats, red foxes, wolves, beech martens), lagomorphs, and humans [1, 3].

The primary vector for the eyeworm in Europe is the male fruitfly *Phortica variegata*, which feeds on lacrimal secretions [8]. Adult female worms release first-stage larvae (L1) into the host’s lacrimal secretions. When the vector feeds on lacrimal secretions, it ingests the L1 stage larvae, which undergo two moults in the fly’s digestive tract before becoming infective third-stage larvae (L3). This process is temperature-dependent and typically takes 14–21 days. The male fly transmits the L3 larvae to a new host during a subsequent feeding. The larvae complete their development into adult worms within the conjunctival sac [9]. Vector activity peaks from spring to late autumn, with optimal conditions at 20–25 °C and 50–70% relative humidity [8].

Clinical signs of *T. callipaeda* infection can vary from mild to severe. In canine thelaziosis conjunctivitis, epiphora, purulent discharge, blepharospasm, keratitis, blepharitis, chemosis, follicular conjunctivitis, and corneal ulceration have been reported [1]. Management typically involves mechanical removal of adult nematodes and pharmacological intervention. Anthelmintic agents are essential for eliminating larvae and preventing recurrence [10].

Human thelaziosis presents mild to severe symptoms, including foreign body sensation, photophobia, lacrimation, epiphora, exudative conjunctivitis, keratitis, and corneal ulcers. The primary treatment method involves mechanical removal of the nematodes under local anaesthesia. Topical and systemic antimicrobials and anti-inflammatory agents are sometimes administered to manage secondary infection and inflammation [11–13].

## Case presentation

In late November 2024, a 6-year-old female spayed Swiss White Shepherd dog presented to the Small Animal Clinic of Estonian University of Life Sciences with unilateral ocular discharge and conjunctival hyperaemia. Prior treatment with fusidic acid eye drops (administered twice daily for 1 week) had not led to clinical improvement. Otherwise, the dog was healthy.

The dog had a travel history from June to August 2024, visiting Latvia, Lithuania, Poland, the Czech Republic, Austria, Italy, Switzerland, Liechtenstein, Germany, and Sweden. The dog received monthly spot-on ectoparasitic treatment with imidacloprid-permethrin during this period. In October, a single dose of prophylactic deworming treatment (milbemycin oxime/praziquantel) was administered to the dog. Ocular signs were noted by the owner in mid-November.

**Fig. 1:**
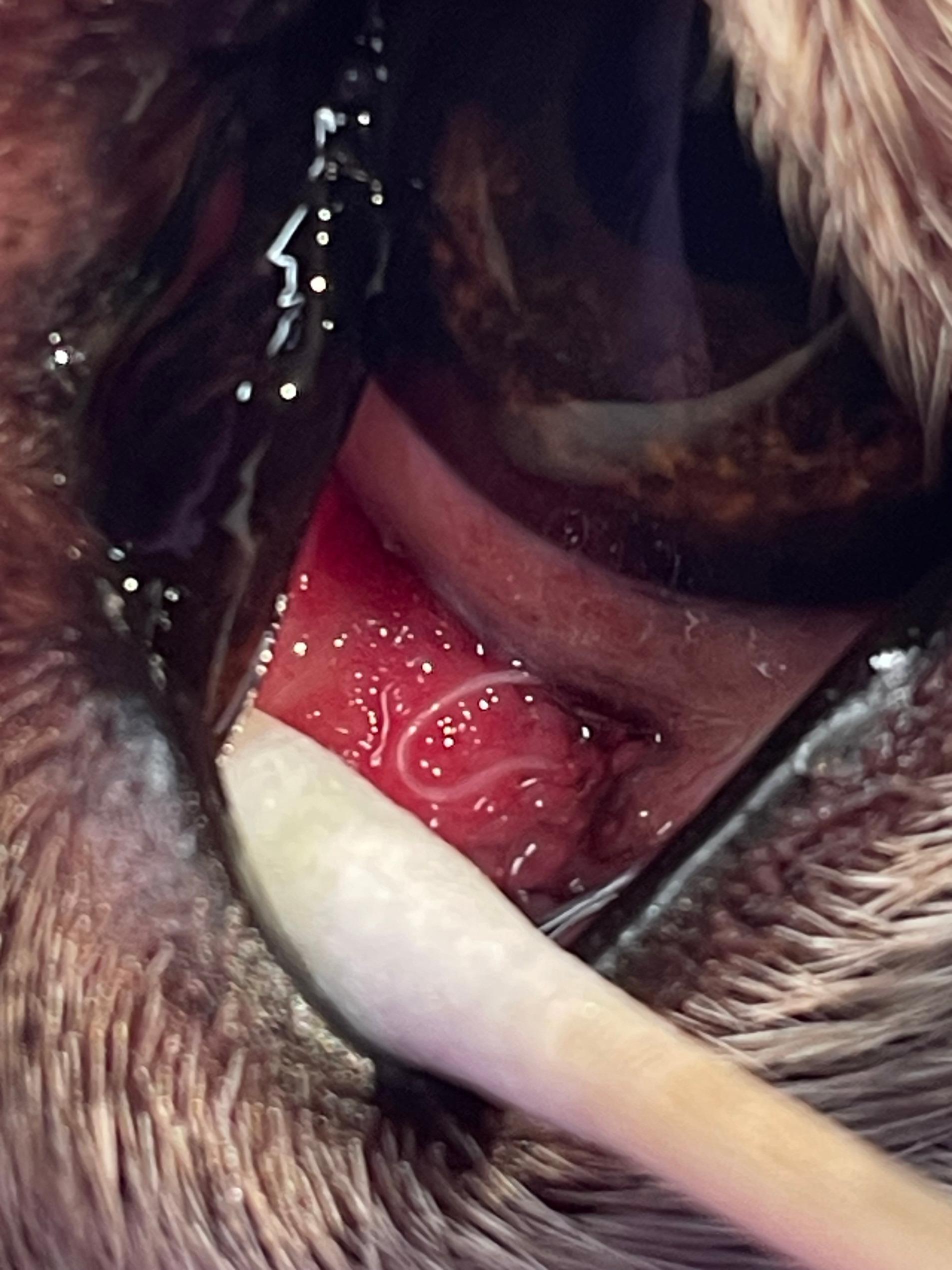
An adult *Thelazia callipaeda* worm visible under the third eyelid of a Swiss White Shepherd dog. A single adult Thelazia callipaeda nematode visible beneath the third eyelid of the left eye during ophthalmological examination of a 6-year-old Swiss White Shepherd dog

Ophthalmological examination revealed hyperaemic conjunctiva in the left eye, slight mucopurulent discharge, and follicular conjunctivitis. Multiple motile, thread-like parasites were observed on the inferior conjunctival fornix and under third eyelid (Fig. 1). A total of eight worms were removed under local anaesthesia using cotton swabs. The specimens were preserved in 80% ethanol and 0.9% sodium chloride solution for molecular identification. The right eye showed no pathological changes and no nematodes were detected.

A clinical examination was otherwise unremarkable. Blood samples were collected for serum biochemical and haematology analysis. Biochemistry revealed mild hypernatremia (164 mmol/L), while haematology parameters were within the reference interval. A peripheral blood smear was examined microscopically to screen for concurrent filarial infections; however, none were observed. Additionally, two patient-side antigen detection tests were performed: Angio Detect^®^ (IDEXX, Westbrook, Maine, USA) and SNAP^®^4Dx^®^ (IDEXX, Westbrook, Maine, USA). Both tests yielded negative results.

The dog was treated with two oral doses of milbemycin oxime/praziquantel, administered 1 week apart. Ocular management consisted of regular cleaning with 0.9% sodium chloride solution and lubrication using a hyaluronate-enriched, carbomer-based ophthalmic gel administered 3–4 times daily, which was continued until the next visit.

A follow-up ophthalmic examination in early December revealed a slight decrease in aqueous tear production on the Schirmer tear test in both eyes (left eye: 12 mm/min; right eye: 10 mm/min). Mild conjunctival hyperaemia and follicular conjunctivitis were noted on the left eye, but no parasites were observed. Ocular cleaning with 0.9% sodium chloride and lubrication continued twice daily until the next follow-up visit. A final examination in January 2025 showed normal Schirmer tear test values in both eyes, and the conjunctivitis had resolved.

Two worm samples were subjected to species identification by using molecular genetic analysis. DNA was extracted using the High Pure PCR Template Preparation Kit (Roche Diagnostics, Mannheim, Germany), following the manufacturer’s protocols. For PCR and sequencing, new primers were designed (The2F GATTGGTGGTTTTGGTAATTGGA and The2R GTACGAGTATCAATATCCAAACCTGCA) that amplified a 697 bp fragment of the mitochondrial DNA cytochrome oxidase I (*cox1*) gene. The analysis yielded two identical sequences of 637 bp. Species identification was conducted by similarity search with nBLAST, which retrieved 21 sequences of *T. callipaeda* from various countries, exhibiting 100% sequence coverage and similarity with the sequences from Estonia. Thus, the species was unequivocally identified as *T. callipaeda*. The *cox1* sequences were submitted to GenBank (accession number PX498064 and PX498065).

### Discussion and conclusions

This report documents the first confirmed case of *T. callipaeda* infection in a dog in Estonia. Given the patient’s travel history through multiple endemic and emerging-risk countries [1, 2, 10], the infection was almost certainly acquired abroad. However, this case underscores the potential for introduction and establishment of *T. callipaeda* in Estonia, particularly if competent vectors such as *P. variegata* are present or become established due to climate change or anthropogenic factors.

Ecological niche models have been used to predict the distribution of *P. variegata*, the vector of *T. callipaeda*, across Europe and the United Kingdom. These models suggest that large areas in Europe are suitable for the vector, highlighting the potential for the spread of *T. callipaeda* into non-endemic regions [14]. Although the vector typically inhabits warmer, oak-rich woodlands in Southern and Central Europe, rising temperatures and vector adaptability may allow its expansion further north. Poland is currently the northeastern limit of reported *T. callipaeda* infection in Europe [7], suggesting that Estonia could be at risk should favourable environmental conditions arise.

The clinical signs observed in this case – unilateral conjunctivitis, mucopurulent discharge, and follicular conjunctivitis – are consistent with previous descriptions in both natural and experimental infections [15]. Awareness of the parasitic aetiology is especially important in non-endemic regions, where *T. callipaeda* may not be considered among differential diagnoses of conjunctivitis. Obtaining a thorough travel history remains crucial for early diagnosis and appropriate management.

Several antiparasitic agents have been evaluated for their efficacy against *T. callipaeda*. In Europe, licensed treatments for canine thelaziosis include a topical spot-on formulation containing moxidectin 2.5% (combined with imidacloprid 10%), as well as an oral formulation of milbemycin oxime. Moxidectin-based products have shown high efficacy in treating and preventing *T. callipaeda* infection [15, 16]. However, adverse weather conditions, such as wet weather causing damp haircoat, may reduce the effectiveness of topical applications [17]. In such cases, oral milbemycin oxime administered at a minimum dose of 0.5 mg/kg offers a practical alternative, particularly for dogs that are difficult to handle for ocular or topical treatments [16].

In the present case, only a single dose of milbemycin oxime was administered in November, and the absence of a follow up dose after one week may have limited the treatment’s success [10, 16].

Prevention plays a critical role in managing the risk of infection during traveling from non-endemic regions to endemic areas. Several studies have been assessing the efficacy of various formulations for prevention. Monthly administration of combined treatments, such as sarolaner/moxidectin/pyrantel or milbemycin oxime/afoxolaner, has demonstrated 100% efficacy in preventing *T. callipaeda* infection [18, 19]. These formulations offer also broad-spectrum control against other parasitic diseases as they are licensed for the treatment and/or prevention of multiple canine parasitoses. Preventive antiparasitic treatment should be routinely recommended for pets traveling abroad.

Further research is warranted to determine the possible distribution of *P. variegata* in Estonia, as well as the potential for autochthonous transmission of *T. callipaeda*. Enhanced surveillance and One Health collaboration among veterinarians, entomologists, wildlife biologists and the public health sectors will be critical to address this emerging parasitic threat.

## Data Availability

The datasets used and/or analysed during the current study are available from the corresponding author on reasonable request.

## Declarations

### Prior publication

Data have not been published previously.

## References

[1] Do Vale B, Lopes AP, Da Conceição Fontes M, Silvestre M, Cardoso L, Coelho AC. Systematic review on infection and disease caused by *Thelazia callipaeda* in Europe: 2001–2020. Parasite. 2020;27. 10.1051/parasite/2020048. 10.1051/parasite/2020048 PMC7526429 32996882

[2] Vieira L, Rodrigues FT, Costa Á, Diz-Lopes D, Machado J, Coutinho T, et al. First report of canine ocular thelaziosis by *Thelazia callipaeda* in Portugal. Parasit Vectors. 2012;5. 10.1186/1756-3305-5-124. 10.1186/1756-3305-5-124 PMC3431252 22720837

[3] Tasić-Otašević S, Gabrielli S, Trenkić-Božinović M, Petrović A, Gajić B, Colella V, et al. Eyeworm infections in dogs and in a human patient in Serbia: A One Health approach is needed. Comp Immunol Microbiol Infect Dis. 2016;45:20–2. 10.1016/j.cimid.2016.01.003. 27012916 10.1016/j.cimid.2016.01.003

[4] Diakou A, Di Cesare A, Tzimoulia S, Tzimoulias I, Traversa D. *Thelazia callipaeda* (Spirurida: Thelaziidae): first report in Greece and a case of canine infection. Parasitol Res. 2015;114:2771–5. 10.1007/s00436-015-4457-4. 25843574 10.1007/s00436-015-4457-4

[5] Farkas R, Takács N, Gyurkovszky M, Henszelmann N, Kisgergely J, Balka G, et al. The first feline and new canine cases of *Thelazia callipaeda* (Spirurida: Thelaziidae) infection in Hungary. Parasit Vectors. 2018;11. 10.1186/s13071-018-2925-2. 10.1186/s13071-018-2925-2 PMC5993998 29884211

[6] Jirků M, Kuchta R, Gricaj E, Modrý D, Pomajbíková KJ. Canine thelaziosis in the Czech Republic: The northernmost autochthonous occurrence of the eye nematode *Thelazia callipaeda* Railliet et Henry, 1910 in Europe. Folia Parasitol (Praha). 2020;67:010. 10.14411/FP.2020.010. 10.14411/fp.2020.010 32367814

[7] Rolbiecki L, Izdebska JN, Franke M, Iliszko L, Fryderyk S. The vector-borne zoonotic nematode *Thelazia callipaeda* in the eastern part of Europe, with a clinical case report in a dog in Poland. Pathogens. 2021;10:1–7. 10.3390/pathogens10010055. 10.3390/pathogens10010055 PMC7826724 33435395

[8] Otranto D, Brianti E, Cantacessi C, Lia RP, Máca J. The zoophilic fruitfly *Phortica variegata*: Morphology, ecology and biological niche. Med Vet Entomol. 2006;20:358–64. 10.1111/j.1365-2915.2006.00643.x. 17199746 10.1111/j.1365-2915.2006.00643.x

[9] Pombi M, Marino V, Jaenike J, Graham-Brown J, Bernardini I, Lia RP, et al. Temperature is a common climatic descriptor of lachryphagous activity period in *Phortica variegata* (Diptera: Drosophilidae) from multiple geographical locations. Parasit Vectors. 2020;13. 10.1186/s13071-020-3955-0. 10.1186/s13071-020-3955-0 PMC7029543 32070408

[10] Marino V, Gálvez R, Mascuñán C, Domínguez I, Sarquis J, Montoya A, et al. Update on the treatment and prevention of ocular thelaziosis (*Thelazia callipaeda*) in naturally infected dogs from Spain. Int J Parasitol. 2021;51:73–81. 10.1016/j.ijpara.2020.08.007. 33091413 10.1016/j.ijpara.2020.08.007

[11] Otranto D, Dutto M, Human Thelaziasis E. Emerg Infect Dis. 2008;14(4):647–9. 10.3201/eid1404.071205. 18394285 10.3201/eid1404.071205 PMC2570937

[12] Otranto D, Traversa D. *Thelazia* eyeworm: an original endo-and ecto-parasitic nematode. Trends Parasitol. 2005;21:1–4. 10.1016/j.pt.2004.10.008. 15639731 10.1016/j.pt.2004.10.008

[13] Bonilla-Aldana DK, Bonilla-Aldana JL, Acosta-España JD, Sah R, Rodriguez-Morales AJ. Thelaziasis in humans: A systematic review of reported cases. New Microbes New Infect. 2025. 10.1016/j.nmni.2025.101599. 40519211 10.1016/j.nmni.2025.101599 PMC12166799

[14] Palfreyman J, Graham-Brown J, Caminade C, Gilmore P, Otranto D, Williams DJL. Predicting the distribution of *Phortica variegata* and potential for *Thelazia callipaeda* transmission in Europe and the United Kingdom. Parasit Vectors. 2018;11. 10.1186/s13071-018-2842-4. 10.1186/s13071-018-2842-4 PMC5924467 29703231

[15] Otranto D, Colella V, Crescenzo G, Solari Basano F, Nazzari R, Capelli G, et al. Efficacy of moxidectin 2.5% and imidacloprid 10% in the treatment of ocular thelaziosis by *Thelazia callipaeda* in naturally infected dogs. Vet Parasitol. 2016;227:118–21. 10.1016/j.vetpar.2016.07.035. 27523947 10.1016/j.vetpar.2016.07.035

[16] Motta B, Schnyder M, Solari Basano F, Nägeli F, Nägeli C, Schiessl B, et al. Therapeutic efficacy of milbemycin oxime/praziquantel oral formulation (Milbemax^®^) against *Thelazia callipaeda* in naturally infested dogs and cats. Parasit Vectors. 2012;5:85. 10.1186/1756-3305-5-85. 22541136 10.1186/1756-3305-5-85 PMC3356600

[17] Bianciardi P, Otranto D. Treatment of dog thelaziosis caused by *Thelazia callipaeda* (Spirurida, Thelaziidae) using a topical formulation of imidacloprid 10% and moxidectin 2.5%. Vet Parasitol. 2005;129:89–93. 10.1016/j.vetpar.2004.12.020. 15817208 10.1016/j.vetpar.2004.12.020

[18] Bezerra-Santos MA, Mendoza-Roldan JA, Sgroi G, Lia RP, Venegoni G, Solari Basano F, et al. Efficacy of a formulation of sarolaner/moxidectin/pyrantel (Simparica Trio^®^) for the prevention of *Thelazia callipaeda* canine eyeworm infection. Parasit Vectors. 2022;15. 10.1186/s13071-022-05501-6. 10.1186/s13071-022-05501-6 PMC9575252 36244989

[19] Lebon W, Guillot J, Álvarez MJ, Antonio Bazaga J, Cortes-Dubly ML, Dumont P, et al. Prevention of canine ocular thelaziosis (*Thelazia callipaeda*) with a combination of milbemycin oxime and afoxolaner (Nexgard Spectra^®^) in endemic areas in France and Spain. Parasite. 2019;26. 10.1051/parasite/2019001. 10.1051/parasite/2019001 PMC6333103 30644355

